# Device‐Measured 24‐h Movement Behaviors and Arterial Intima‐Media Thickness in Young Adults

**DOI:** 10.1111/sms.70387

**Published:** 2026-10-24

**Authors:** Kristin Suorsa, Tomi Laitinen, Jarkko Heiskanen, Tuija Leskinen, Yaxing Meng, Jesse Pasanen, Suvi Rovio, Juhani S. Koskinen, Juha Mykkänen, Olli J. Heinonen, Jorma Viikari, Tapani Rönnemaa, Harri Niinikoski, Olli Raitakari, Sari Stenholm, Katja Pahkala

**Affiliations:** ^1^ Department of Public Health University of Turku and Turku University Hospital Turku Finland; ^2^ Centre for Population Health Research University of Turku and Turku University Hospital Turku Finland; ^3^ Research Centre of Applied and Preventive Cardiovascular Medicine University of Turku Turku Finland; ^4^ Paavo Nurmi Centre & Unit for Health and Physical Activity University of Turku Turku Finland; ^5^ Department of Medicine, University of Turku and Division of Medicine Turku University Hospital Turku Finland; ^6^ Department of Medicine Satakunta Central Hospital Pori Finland; ^7^ Department of Pediatrics and Adolescent Medicine, Turku University Hospital University of Turku Turku Finland; ^8^ Department of Clinical Physiology and Nuclear Medicine Turku University Hospital, University of Turku Turku Finland; ^9^ Research Services Turku University Hospital and University of Turku Turku Finland

**Keywords:** accelerometry, compositional data analysis, intima‐media thickness, physical activity, sedentary behavior, sleep, ultrasonography

## Abstract

Physical activity, including light physical activity (LPA) and moderate‐to‐vigorous physical activity (MVPA), sedentary behavior (SED), and sleep, often referred to as “24‐h movement behaviors”, are modifiable behaviors that may be associated with arterial intima‐media thickness (IMT). However, their combined associations with arterial IMT, especially aortic IMT, where cardiometabolic risk burden may be reflected at an earlier age, have not been studied before. This study examined how 24‐h movement behaviors associate with aortic and carotid IMT among young adults. Participants (*n* = 327) were healthy young adults (mean age 26.1 years, SD 0.2) from the Special Turku Coronary Risk Factor Intervention Project (STRIP). 24‐h movement behaviors were measured using a wrist‐worn accelerometer for 1 week. Aortic and carotid IMT were measured with ultrasonography. Associations between 24‐h movement behaviors and aortic and carotid IMT were analyzed using compositional data analysis and compositional isotemporal substitution analysis, adjusting for sex, education, smoking, and distribution of dietary fats. Descriptive model‐based estimations indicated lower aortic IMT values for compositions with a higher proportion of LPA. However, theoretical time reallocations between LPA and other behaviors did not indicate significant differences in aortic IMT (e.g., 30 min from sleep to LPA, −0.005 mm [95% CI −0.020−0.010 mm], from SED to LPA, −0.006 mm [95% CI −0.018−0.006], and from MVPA to LPA, −0.008 mm [95% CI −0.038−0.021]). No associations were found for carotid IMT either. No apparent associations were observed between 24‐h movement behaviors and arterial IMT among young adults.

## 1 Introduction

Atherosclerotic cardiovascular diseases are a major public health concern worldwide [1, 2], and their origins can be traced back to early life [3]. Early vascular remodeling and morphologic adaptations can be found before cardiovascular diseases manifest, for example, using ultrasonically measured arterial intima‐media thickness (IMT) [4, 5]. Previous studies have suggested that cardiometabolic risk burden, for example, higher body mass index (BMI), systolic blood pressure, blood cholesterol concentration, inflammation, and insulin resistance, is reflected at an earlier age on aortic IMT than on the most commonly assessed carotid IMT [6, 7, 8, 9]. Nevertheless, systolic blood pressure has been observed to be one of the few common determinants of both aortic and carotid IMT already at a young age [8, 10, 11, 12].

Physical activity, sedentary behavior (SED), and sleep, often referred to as 24‐h movement behaviors, are modifiable behaviors that may be associated with the development or progression of IMT. We have previously shown in the Special Turku Coronary Risk Factor Intervention Project (STRIP) study that self‐reported leisure time physical activity was favorably associated with aortic IMT in adolescence [13]. Survey‐based studies among adults have indicated that those having low physical activity levels [14], high sitting time (while watching TV) [15], and short sleep [16] have the highest carotid IMT, but associations may be sex‐dependent [14]. However, device‐based data are needed to overcome limitations of surveys, as self‐reported measures are prone to recall and information bias, and they are unable to capture all movement and non‐movement behaviors throughout the 24‐h day [17].

To date, there are only a few device‐based studies examining associations between physical activity, SED, and sleep in isolation from each other and arterial IMT, and evidence in young adults is even scarcer. Moreover, these few studies have focused only on a more traditional target of IMT measurement, the carotid artery, potentially due to its rather superficial location and thus, easier visualization compared with the abdominal aorta [18]. Studies among adolescents have reported mixed results, as no cross‐sectional or longitudinal associations were found for moderate‐to‐vigorous physical activity (MVPA) [19, 20], while cumulative exposure to LPA and MVPA was associated with lower carotid IMT, as well as lower odds of progressive carotid IMT worsening (defined as being above the 90th percentile) [21]. Among middle‐aged and older adults, some studies have reported favorable cross‐sectional and longitudinal associations for LPA and MVPA [22, 23, 24], and unfavorable for SED [24, 25], while other studies have not found associations [26, 27]. Evidence from device‐based studies among middle‐aged and older adults has suggested that especially short sleep (< 5 h) associates with higher carotid IMT [28, 29], and that longer sleep associates with lower carotid IMT among men [30], but an indication of a possible U‐shaped association has also been found [29]. These prior findings suggest that all 24‐h movement behaviors should be considered when examining vascular health. However, we are not aware of previous studies that have examined the combined associations of 24‐h movement behaviors with arterial IMT.

Given that sleep, SED, and physical activity are fixed to the 24‐h day, time spent in one behavior cannot be increased without reducing at least one other behavior, making them codependent on each other. Health associations may depend on which behavior is replaced; for instance, whether increased physical activity replaces SED or sleep. Commonly used methodologies to take into account the codependency between 24‐h movement behaviors and to model health effects of time reallocations between movement behaviors are compositional data analysis (CoDA) [31, 32] and compositional isotemporal substitution analysis [33].

To fill the gaps in previous studies, we examined the association between the 24‐h movement behaviors and aortic and carotid IMT among young adults using CoDA and compositional isotemporal substitution analysis.

## 2 Methods

### 2.1 Study Population

The Special Turku Coronary Risk Factor Intervention Project (STRIP) study is a prospective, randomized controlled trial aiming to prevent atherosclerosis beginning in infancy [34]. A detailed description of the study design has been reported elsewhere [34, 35]. In brief, families of 5‐month‐old infants, born between July 1989 and December 1991, were recruited at well‐baby clinics in Turku, Finland, by the clinic nurses. The intervention group received dietary intervention between the ages of 7 months and 20 years, while the control group received the basic health education given at Finnish well‐baby clinics and school health care. In this study, the participants were treated as a single cohort, irrespective of their STRIP study group allocation (intervention/control).

This study is based on the first post‐intervention follow‐up, which was conducted between April 2015 and January 2018, when participants were 26 years old. Altogether, 1072 study participants were invited to participate in a clinical study visit, which included blood sampling and ultrasound measurements of arterial IMT [36, 37]. Formation of the study sample is illustrated in Data S1. Of the invited participants, 546 attended the study visit, of whom 402 (74%) participated in accelerometer measurement. The remaining participants either were unwilling to participate in the accelerometer measurement (*n* = 58), did not provide information for accelerometer delivery (*n* = 2), or did not wear the accelerometer (*n* = 84, including 2 participants with Type 1 diabetes). Among the 402 participants with accelerometer data, we excluded those with Type 1 diabetes (*n* = 4), those with fewer than four valid measurement days of at least 10 h of waking wear time per day based on the commonly used criteria [38] (*n* = 58), device malfunction (*n* = 5), and participants with missing data on aortic and/or carotid IMT (*n* = 8), leaving 327 participants in the analytical sample.

### 2.2 Assessment of Arterial Intima‐Media Thickness

IMT of the abdominal aorta and common carotid arteries was assessed using a GE Vivid E9 ultrasound system (GE Vingmed Ultrasound A/S, Horten, Norway), described in detail in Data S1.

### 2.3 Assessment of 24‐h Movement Behaviors

A triaxial ActiGraph wActiSleep‐BT accelerometer (ActiGraph, Pensacola, Florida, US) was used to estimate the 24‐h movement behaviors, that is, sleep, SED, LPA, and MVPA. The accelerometers were initialized to record at 80 Hz. A member of the study staff gave the accelerometers to participants during the study visit and guided the participants on the use of the accelerometers. The participants were instructed to wear the accelerometer on their non‐dominant wrist for the following 8 days at all times, 24 h/day, including during water‐based activities such as swimming, but to remove it for sauna. In an accompanying log, the participants were asked to record the date, wake‐up time, and bedtime on each measurement day. After the measurement period, the participants returned the accelerometer and the log by mail. Data from the accelerometers were downloaded in the ActiLife software, version 6.13 (ActiGraph, Pensacola, Florida, US). Accelerometer data were collected between April 2015 and March 2018.

The accelerometer data were processed using previously reported methods [39]. Briefly, the R package GGIR version 1.7‐1 [40] was used to estimate 24‐h movement behaviors, after detecting and excluding non‐wear time. The sleep period was detected based on the combination of the daily logs and the algorithm of the GGIR package [41], so that sleep onset and wake‐up time were estimated within the time in bed period reported in the daily logs during which there was no change larger than 5° in the arm angle over at least 5 min. The sleep period was then defined as the interval between estimated sleep onset and wake‐up time. Thus, the sleep period included wake after sleep onset time but excluded sleep onset latency, that is, the time required to fall asleep. Hereafter, the term “sleep” refers to the sleep period estimate. Waking‐time SED, LPA, and MVPA were defined using the threshold values of < 44.8 m*g*, at least 44.8 m*g* but < 100.6 mg, and ≥ 100.6 mg, respectively [42, 43].

The measurement day was determined from each measurement day's sleep onset time to the next measurement day's sleep onset time. The analyses were restricted to valid measurement days with at least 10 h of waking wear time. No specific restrictions were made regarding night duration. The average duration of a valid measurement day was 24.0 h (SD 0.55 h), indicating very good compliance for the 24‐h measurement. Weekly compositional means of time spent in each behavior were calculated and scaled to be proportional to a 24‐h day.

### 2.4 Participant Sociodemographic and Health‐Related Characteristics

Sex and level of education were obtained from a self‐administered questionnaire. Education was classified as lower education (upper secondary education or lower) and higher education (ongoing or completed university or university of applied sciences education). Participants reporting smoking at least once a day were regarded as regular smokers. The distribution of dietary fats was examined by using a 4‐day food record and defined as a ratio of monounsaturated fatty acids (MUFA) and polyunsaturated fatty acids (PUFA) to saturated fatty acids (SFA) ((MUFA+PUFA)/SFA) [44].

For the comparison of the study participants to non‐participants of accelerometer and IMT measurements with respect to health‐related characteristics, self‐reported leisure time physical activity was calculated using questionnaire‐based frequency, duration, and intensity of habitual leisure time physical activity. Leisure time physical activity was calculated as a multiple of the resting metabolic rate (metabolic equivalent [MET] h/week) by multiplying the frequency, mean duration, and mean intensity of weekly leisure time physical activity. Self‐reported daily sitting time was estimated by summing up self‐reported time spent sitting at the office, while watching TV or using a computer, in a vehicle, or in other contexts. Self‐reported sleep period per 24‐h day was also inquired about in the questionnaire. Waist circumference (cm), body mass index (BMI; kg/m^2^), and diastolic and systolic blood pressure (mmHg) were measured by a study nurse at the clinical study visit [36]. Concentrations of serum lipids (total cholesterol, low‐density lipoprotein [LDL] cholesterol, high‐density lipoprotein [HDL] cholesterol, triglycerides), homeostasis model assessment of insulin resistance (HOMA‐IR), and high‐sensitivity C‐reactive protein (hs‐CRP) were analyzed using standard methods [36].

### 2.5 Statistical Analysis

The characteristics of the study population are presented as means (SDs) for continuous variables, and as frequencies and percentages for categorical variables. For categorical variables, the *χ* ^2^‐test was used, and for continuous variables, the Student's *t‐*test was applied. Given that the intervention and control groups did not differ in terms of 24‐h movement behaviors (*p* = 0.95) or in aortic (*p* = 0.91) and carotid IMT (*p* = 0.23), and that no significant interaction between the groups was observed (for aortic IMT *p* = 0.62, carotid IMT *p* = 0.15), the participants were treated as a single cohort, irrespective of their STRIP study group allocation. The present study population (*n* = 327) was also compared with non‐participants in the accelerometer and IMT measurements (*n* = 213; participants with Type 1 diabetes, *n* = 6, excluded from the comparison group) with respect to sociodemographic and health‐related characteristics, as well as self‐reported 24‐h movement behaviors.

### 2.6 Compositional Data Analysis

The proportion of time spent in each behavior was treated as compositional data. The analyses were conducted using the packages *compositions* [45], *robCompositions* [46], and *codaredistlm* [47] in R version 4.3.1 [48]. The compositional means were calculated as the component‐wise geometric means of the data and rescaled to sum up to 1440 min. The dataset did not include zero values for weekly means of any of the 24‐h movement behaviors; thus, imputation was not needed.

An isometric logratio (ilr) transformation was used to map the compositional data into real‐valued coordinates, which reduces the dimensionality of the data and allows standard statistical methods to be used [49]. We used *pivot coordinates*, a specific type of *ilr* coordinates, which are a set of *ilrs* where the first coordinate enables one part of the composition (for instance, sleep) to be considered relative to the remaining parts of the composition (i.e., SED, LPA, and MVPA), resulting in four sets of pivot coordinates.

We examined the associations between 24‐h movement behaviors and aortic and carotid IMT using compositional linear regression models. Aortic and carotid IMT values were used as the dependent variables, and 24‐h movement behaviors (expressed as *ilrs*) as the independent variables. Model 1 was adjusted for sex. Model 2 included additional adjustment for education, Model 3 for smoking and the distribution of dietary fats, and Model 4 for BMI, HOMA‐IR, and systolic blood pressure, previously identified as determinants of aortic and carotid IMT in the STRIP study population [8]. All models were repeated for each set of pivot coordinates. The associations were presented as beta coefficients with their 95% confidence intervals (CI). The beta coefficients indicate the change in the dependent variable (e.g., mm) for each one‐unit *ilr* increase, thus pointing out the presence of association. However, effect sizes cannot be directly inferred from the beta coefficients.

To illustrate model‐based variation in aortic and carotid IMT values across 24‐h movement behavior compositions, the previously described sex‐ and education‐adjusted four‐part compositional linear regression model was used to estimate aortic and carotid IMT for simulated 24‐h movement behavior compositions within the range observed in the study sample. The resulting model‐based estimates were visualized using heat map ternary plots. Each ternary plot represents a conditional slice of the four‐part composition, displaying the relative proportions of three movement behaviors while the fourth behavior was held constant at a predefined reference value close to the sample mean composition. The reference values were rounded to practical values for visualization. Estimated IMT values were derived from the complete four‐part composition, including sleep, SED, LPA, and MVPA.

To estimate the cross‐sectional differences in aortic and carotid IMT associated with theoretical time reallocations between 24‐h movement behaviors, a compositional isotemporal substitution model [33] was applied. Systematic reallocations between 24‐h movement behaviors were calculated based on the mean composition of the study population (8.0 h sleep, 12.8 h SED, 1.7 h LPA, and 1.4 h MVPA). The size of the reallocations ranged from −60 min to 60 min in 10‐min increments. These reallocated compositions (e.g., 30 min from SED to LPA, while keeping MVPA and sleep constant) were transformed to *ilrs* using the method described above. The regression‐based coefficients from the sex‐ and education‐adjusted model were then applied to estimate differences in aortic and carotid IMT corresponding to theoretical time reallocations in the 24‐h movement behavior composition. The results are shown as estimated differences in aortic and carotid IMT values with their 95% CIs. When the confidence interval did not cover 0, the difference was considered significant. Moreover, the regression‐based coefficients with a logit link were used to estimate the differences in the odds of elevated aortic and carotid IMT (defined as ≥ 90th percentile by sex for aortic and carotid IMT) associated with theoretical time reallocation of 30 min between 24‐h movement behaviors. When the CI did not cover 1, the odds were considered significant.

To provide insight into potential physiological mechanisms between 24‐h movement behaviors and arterial IMT, we conducted a post hoc analysis examining the associations between 24‐h movement behaviors and blood pressure using compositional linear regression and isotemporal substitution models. As for the IMT outcomes, model 1 was adjusted for sex, model 2 additionally for education, and model 3 for smoking and the distribution of dietary fats. Isotemporal substitution analyses were conducted similarly to those for the IMT outcomes, using the beta coefficients from model 2.

As an additional analysis, given that previous studies have suggested sex differences in the association between physical activity and sleep with carotid IMT [21, 30], we tested the interaction between the 24‐h movement behavior composition (i.e., ilrs) and sex for both aortic and carotid IMT in the model adjusted for sex and education. Moreover, as previous studies have suggested that the association between sleep and cardiovascular health may be U‐shaped [29, 50, 51], we conducted an exploratory effect‐modification analysis by adding an interaction term between the 24‐h movement behavior composition and a three‐level indicator of sleep duration in the model adjusted for sex and education. While prior research has linked very short sleep (< 5 h/night) with higher carotid IMT [28, 29], in our sample, there were only a few participants with < 5 h of sleep. Therefore, sleep duration was categorized according to the latest recommendations on healthy sleep range [52], to (1) “short sleep”, defined as less than 7 h of sleep per night (*n* = 42, 13%), (2) “sufficient sleep”, defined as 7–9 h of sleep per night (*n* = 255, 78%), and (3) “long sleep”, defined as more than 9 h of sleep per night (*n* = 30, 9%). To account for the potential effect of total accelerometer wear time per measurement day (from the previous day's sleep onset time to next day's sleep onset time) on the results, we also conducted an additional analysis in which the sex‐ and education‐adjusted model was further adjusted for mean total accelerometer wear time per measurement day.

## 3 Results

A slight majority of the 26‐year‐old study participants were women (60%) (Table 1). The majority of the participants had higher education (ongoing or completed university or university of applied sciences education, 66%), and only 9% reported smoking. The mean aortic and carotid IMT values were 0.60 mm and 0.46 mm, respectively, and the mean systolic and diastolic blood pressure were 120.0 and 71.6 mmHg. Comparison of the analytical sample with those STRIP study participants who did not provide sufficient accelerometer or arterial IMT data showed no differences in self‐reported leisure‐time physical activity, sitting time, or aortic or carotid IMT (Data S3). Moreover, no differences were observed in education or most health‐related characteristics. However, the present study population had a higher proportion of women and tended to have longer self‐reported sleep duration and lower systolic blood pressure.

**TABLE 1:** Characteristics of the study participants at age 26 years (*n* = 327).

	All (*n* = 327)
Women, *n* (%)	195 (60)
Education, *n* (%)^a^	
Lower	105 (32)
Higher	217 (66)
Current smoking, *n* (%)^a^	29 (9)
Distribution of dietary fats^b^, mean (SD)	1.6 (0.6)
Compositional mean of sleep, SED, LPA, MVPA, h	8.0, 12.8, 1.7, 1.4
Aortic IMT, mean (SD), mm	0.60 (0.15)
Carotid IMT, mean (SD), mm	0.46 (0.05)
BMI, mean (SD), kg/m^2^	24.4 (4.1)
Waist circumference, mean (SD), cm	80.3 (11.4)
Systolic blood pressure, mean (SD), mmHg	120.0 (10.4)
Diastolic blood pressure, mean (SD), mmHg	71.6 (6.8)
Total cholesterol, mean (SD), mmol/L	4.6 (0.9)
LDL cholesterol, mean (SD), mmol/L	2.8 (0.8)
HDL cholesterol, mean (SD), mmol/L	1.3 (0.3)
Triglycerides, mean (SD), mmol/L	1.0 (0.5)
HOMA‐IR, mean (SD)	1.7 (1.1)
hs‐CRP, mean (SD), mg/L	2.0 (3.4)
Number of valid accelerometer measurement days, mean (range)	6.6 (4–9)

Abbreviations: BMI = body mass index, HDL = high‐density lipoprotein, HOMA‐IR = homeostasis model assessment of insulin resistance, hs‐CRP = high‐sensitivity C‐reactive protein, IMT = intima‐media thickness, LDL = low‐density lipoprotein, LPA = light physical activity, MVPA = moderate‐to‐vigorous physical activity, SED = sedentary behavior.

^a^ Missing values, *n* = 5 (1.5%). Percentages are based on the total study sample (*n* = 327).

^b^ A ratio of monounsaturated fatty acids (MUFA) and polyunsaturated fatty acids (PUFA) to saturated fatty acids (SFA) ((MUFA+PUFA)/SFA).

To illustrate model‐based variation in estimated aortic and carotid IMT across 24‐h movement behavior compositions within the range observed in the study sample, ternary heat maps representing conditional slices of the four‐part composition were created (Figures 1 and 2). In these visualizations, the omitted behavior was fixed at a predefined reference value corresponding approximately to the sample mean composition. Lower aortic IMT values (green areas) were estimated for compositions with higher proportions of LPA relative to SED (Figure 1A,B) and sleep (Figure 1C), whereas the proportions of MVPA relative to other behaviors did not appear to be related to aortic IMT values (Figure 1A,C,D). In contrast, variation in estimated carotid IMT across different 24‐h movement behavior compositions was considerably smaller than that observed for aortic IMT (0.456–0.464 mm vs. 0.579–0.635 mm, respectively). Lower carotid IMT values were estimated for compositions with higher proportions of LPA and SED in relation to sleep and MVPA (Figure 2C,D).

**FIGURE 1:**
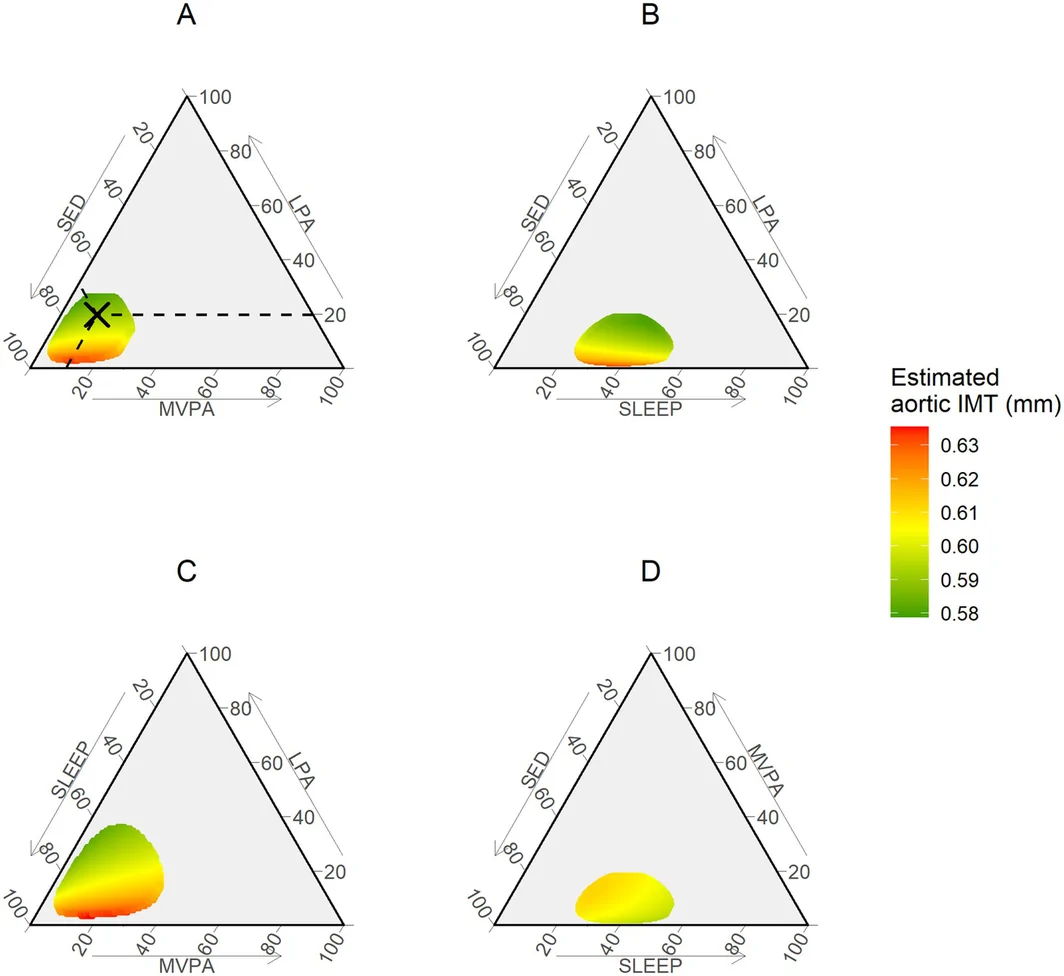
Heat map ternary plots illustrating model‐based estimates of aortic intima‐media thickness (IMT) values for different 24‐h movement behavior compositions within the range observed in the study sample. Estimated aortic IMT values were obtained from a sex‐ and education‐adjusted four‐part compositional regression model including sleep, sedentary behavior (SED), light physical activity (LPA), and moderate‐to‐vigorous physical activity (MVPA). The color scale reflects estimated aortic IMT (mm), with red indicating higher values relative to the sample mean (0.60 mm), yellow values close to the sample mean, and green indicating lower values relative to the sample mean. Each panel represents a conditional slice of the four‐part composition, with the omitted behavior fixed at a predefined reference value corresponding approximately to the sample mean composition. Panel (A) displays the relative proportions of SED, LPA, and MVPA, while sleep was held constant at 480 min. For illustration, point X corresponds to a three‐part subcomposition consisting of 69% SED, 20% LPA, and 11% MVPA. The displayed proportions are relative to the three behaviors shown in the ternary plot and therefore sum to 100%. According to the full four‐part compositional model, the corresponding estimated aortic IMT is 0.59 mm. Panel (B) displays the relative proportions of SED, LPA, and sleep, while MVPA was held constant at 90 min. Panel (C) displays the relative proportions of sleep, LPA, and MVPA, while SED was held constant at 770 min. Panel (D) displays the relative proportions of SED, MVPA and sleep while LPA was held constant at 100 min.

**FIGURE 2:**
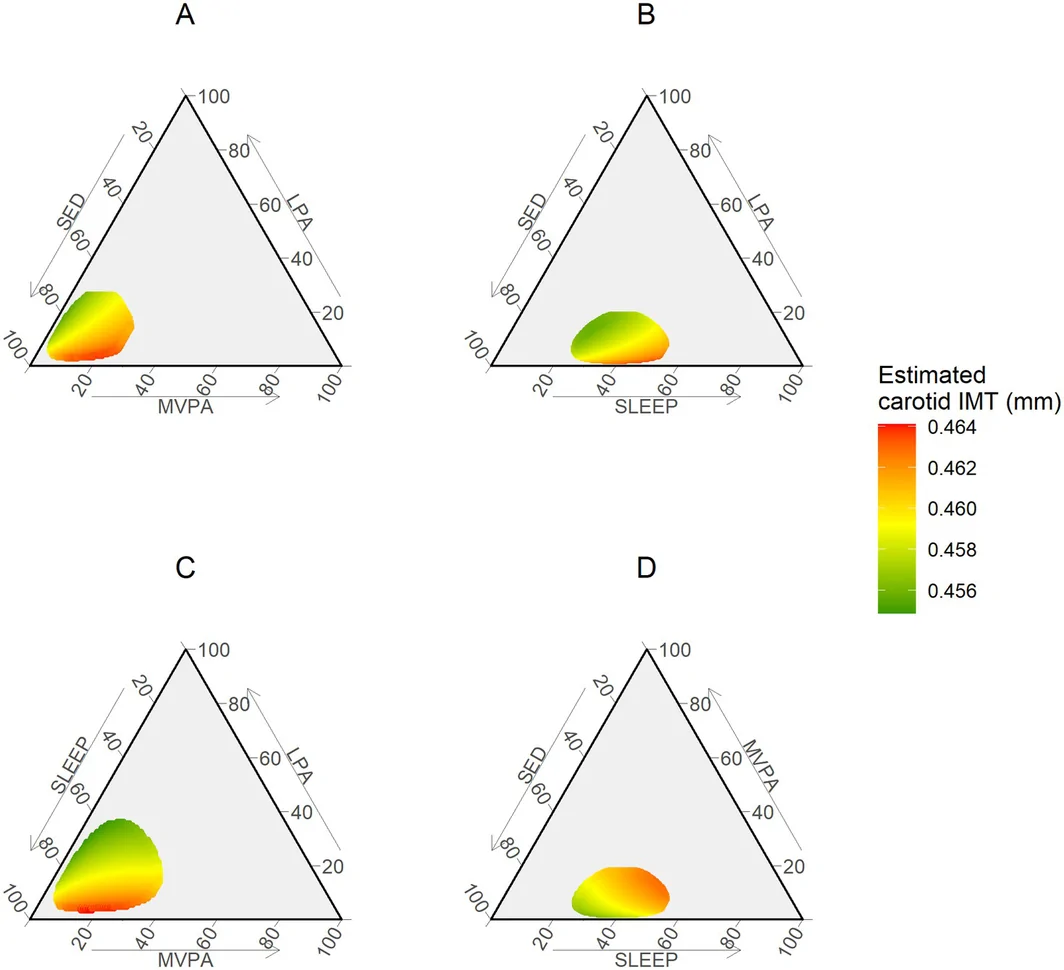
Heat map ternary plots illustrating model‐based estimates of carotid intima‐media thickness (IMT) for 24‐h movement behavior compositions within the range observed in the study sample. Estimated carotid IMT values were obtained from the same four‐part compositional regression model and visualized using the same conditional‐slice approach as described in Figure 1. The color scale reflects estimated carotid IMT (mm), with red indicating higher values relative to the sample mean (0.46 mm), yellow values close to the sample mean, and green indicating lower values relative to the sample mean. Panels (A–D) correspond to the same fixed reference values and movement‐behavior combinations as in Figure 1, with the omitted behavior held constant at the corresponding reference value in each panel.

However, when examining the associations of each 24‐h movement behavior relative to the remaining behaviors (e.g., LPA in relation to sleep, SED, and MVPA) with aortic and carotid IMT, no significant associations were observed (Data S4).

To further examine the associations between theoretical reallocation of time from specific 24‐h movement behavior to another movement behavior (e.g., from SED to LPA) and aortic and carotid IMT, isotemporal substitution analysis was conducted and illustrated in Figures 3 and 4. In general, theoretical time reallocation from other behaviors to LPA tended to be associated with slightly lower estimated aortic IMT, but no significant associations were observed (for instance, reallocation of 30 min from SED to LPA in the sex‐ and education‐adjusted model, estimated cross‐sectional difference in aortic IMT of −0.006 mm [95% CI −0.018−0.006], from sleep to LPA −0.005 mm [95% CI −0.020−0.010], and from MVPA to LPA −0.008 mm [95% CI −0.038−0.021]). Theoretical time reallocations between movement behaviors did not appear to be associated with estimated differences in carotid IMT (Figure 4). Across the examined 30‐min time reallocations, the upper and lower limits of the 95% confidence intervals suggest that differences larger than approximately 0.01 mm could reasonably be excluded. Similarly, theoretical time reallocations between 24‐h movement behaviors were not related to the odds of elevated aortic or carotid IMT (defined as ≥ 90th percentile) (Data S5 and S6).

**FIGURE 3:**
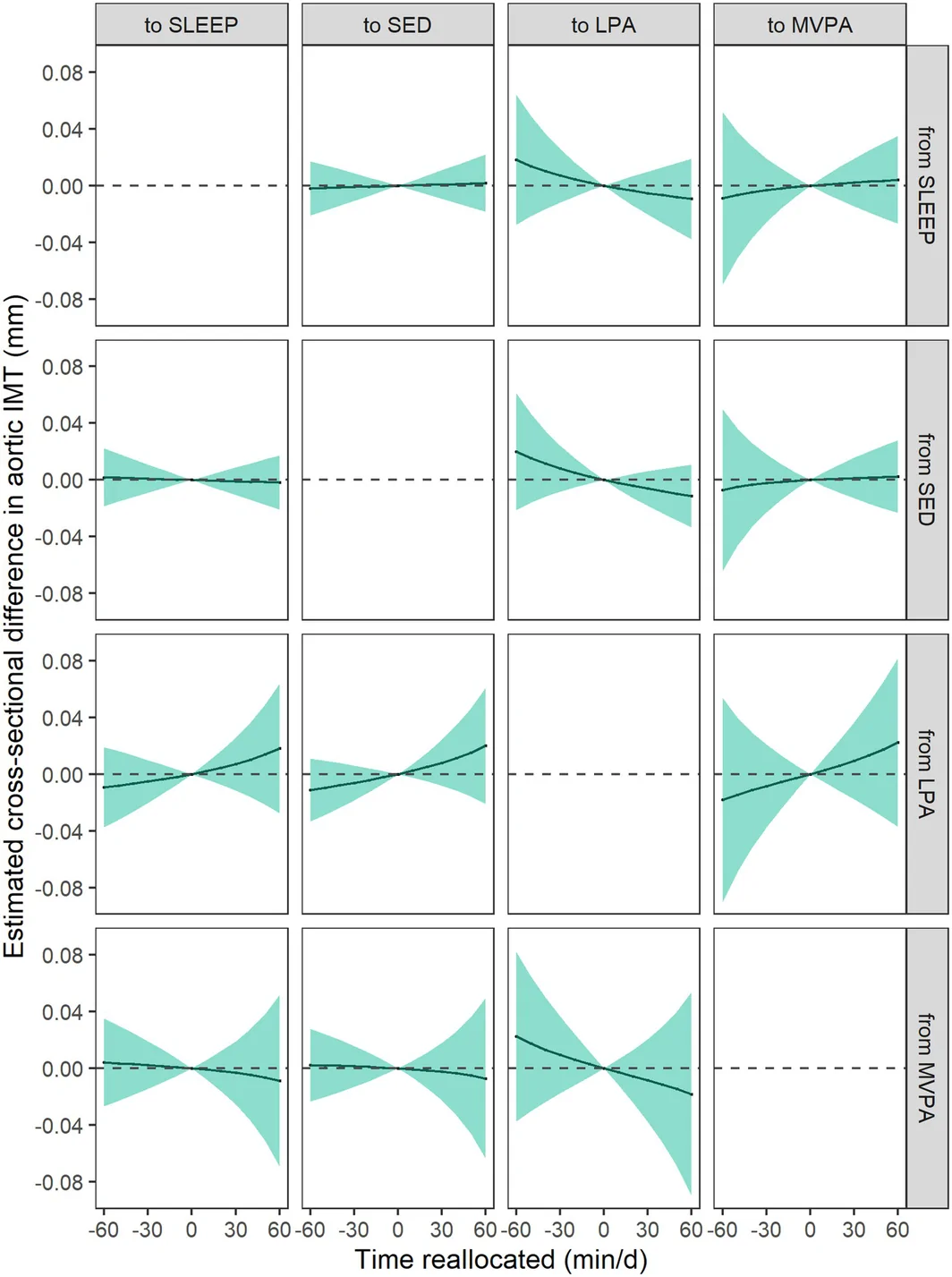
Estimated cross‐sectional differences in aortic intima‐media thickness (IMT) associated with theoretical one‐to‐one reallocations between 24‐h movement behaviors (e.g., from SED to LPA) relative to the mean 24‐h movement behavior composition in the study population (8.0 h sleep, 12.8 h SED, 1.7 h LPA, and 1.4 h MVPA). Estimates were obtained from a sex‐ and education‐adjusted compositional linear regression model. Shaded areas indicate 95% confidence intervals around the estimated differences.

**FIGURE 4:**
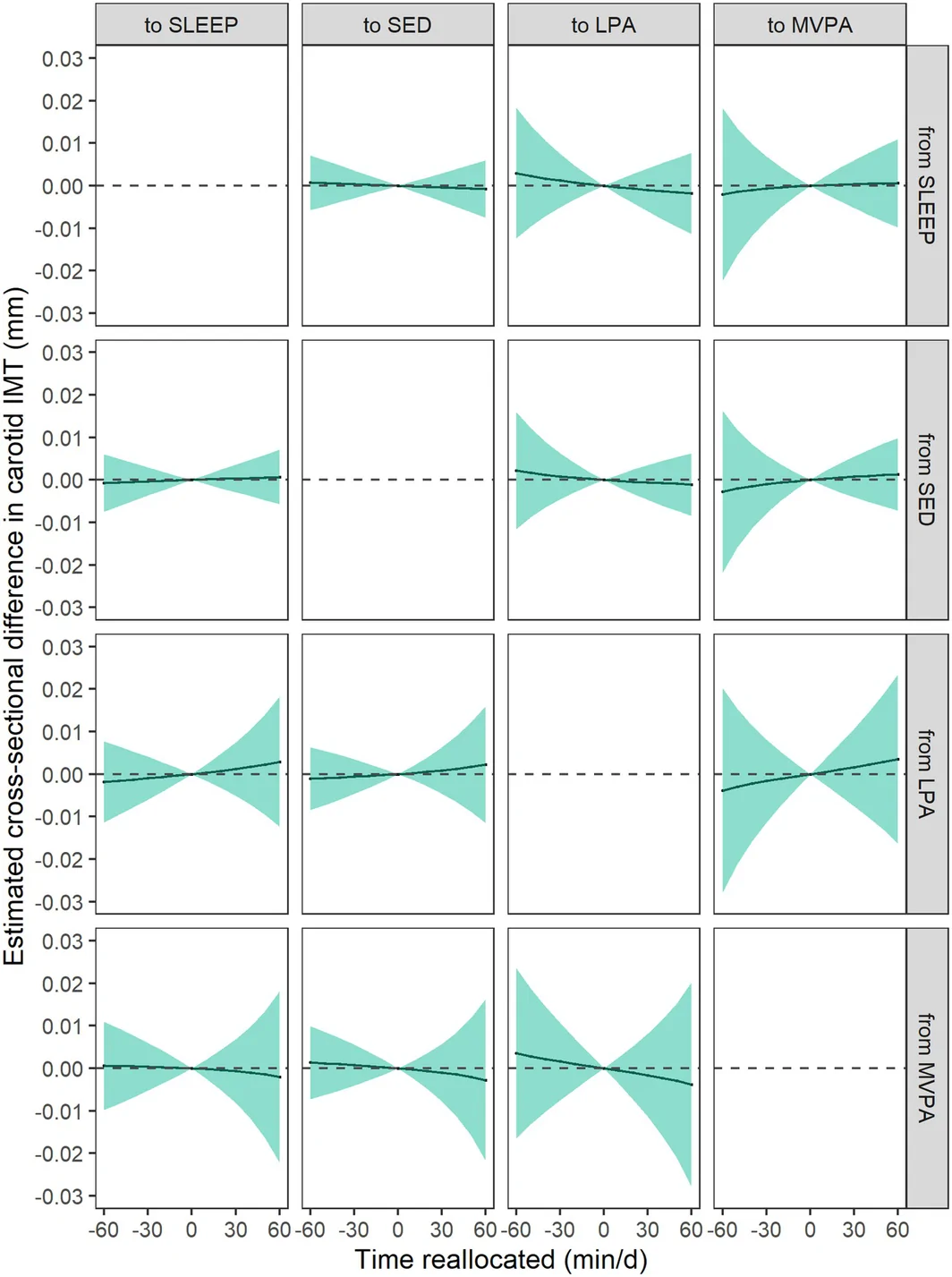
Estimated cross‐sectional differences in carotid intima‐media thickness (IMT) associated with theoretical one‐to‐one reallocations between 24‐h movement behaviors (e.g., from SED to LPA) relative to the mean 24‐h movement behavior composition in the study population (8.0 h sleep, 12.8 h SED, 1.7 h LPA, and 1.4 h MVPA). Estimates were obtained from a sex‐ and education‐adjusted compositional linear regression model. Shaded areas indicate 95% confidence intervals around the estimated differences.

As a post hoc analysis, the associations between 24‐h movement behaviors and blood pressure, an important determinant of arterial IMT, were examined. Higher MVPA relative to the remaining behaviors was associated with higher systolic blood pressure (Data S8). Especially, the theoretical time reallocation from sleep to MVPA was significantly associated with higher systolic blood pressure (for instance, 30 min from sleep to MVPA estimated change 1.05 mmHg [95% CI 0.09–2.00]) (Data S9). A slight trend towards lower systolic blood pressure was also observed with theoretical time reallocation from SED to LPA (30 min from SED to LPA, estimated difference −0.52 mmHg [95% CI −1.20−0.17]) (Data S9). No associations were observed for diastolic blood pressure (Data S8 and S10).

To examine the potential modifying effect of sex, the interaction between the composition of 24‐h movement behaviors and sex was examined. No significant interaction was observed for aortic IMT (*p* = 0.41) or carotid IMT (*p* = 0.27). To examine the potential modifying effect of sleep duration on the association between the 24‐h movement behavior composition and aortic and carotid IMT, the interaction between the composition of 24‐h movement behaviors and sleep category was added to the model. The interaction was not significant for either aortic or carotid IMT (*p* = 0.58 and *p* = 0.59). Additional adjustment for accelerometer wear time per measurement day did not alter the interpretation of the results (Data S7).

## 4 Discussion

Our study addresses previous research gaps by using device‐based measures of all 24‐h movement behaviors and by examining associations for not only carotid but also aortic IMT in a young adult population. In contrast to previous studies, we also accounted for the codependency between 24‐h movement behaviors by applying CoDA methodology. Although there was some indication that higher levels of LPA relative to the remaining movement behaviors may be associated with lower aortic IMT, we did not observe significant associations between 24‐h movement behaviors and arterial IMT among apparently healthy and active 26‐year‐old adults.

As a novel aspect of our study, we report associations between device‐based 24‐h movement behaviors and aortic IMT. Building on pioneering work in the 2000s that identified aortic IMT as the most informative non‐invasive marker of preclinical atherosclerosis at a young age [18], the STRIP study stands out by including aortic IMT assessments from early adolescence, a feature not present in most other cohorts that have focused solely on carotid IMT. Previous studies from the STRIP study cohort have indicated that self‐reported leisure time physical activity, cardiometabolic risk burden, physical fitness, and healthy diet are reflected in aortic, but not carotid, IMT already during adolescence [8, 13, 44, 53]. In the present study, consisting of the STRIP study participants who used accelerometry at age 26, we did not find significant associations between device‐measured LPA or MVPA and aortic or carotid IMT. There was only a slight trend towards higher levels of device‐measured LPA and lower aortic, but not carotid, IMT. As a possible explanation, cardiometabolic risk factors have been reported to correlate more strongly with aortic IMT in adolescents than in young adults, suggesting a greater vascular sensitivity of the aorta during adolescence compared with young adulthood [9]. Further research clarifying how the association between 24‐h movement behaviors and aortic IMT evolves across the life course would provide useful insights. Such information could support the identification of potential periods of higher vascular susceptibility to modifications of 24‐h movement behaviors, when interventions targeting these behaviors may be most effective in slowing aortic IMT progression.

Our study complements existing knowledge on the associations between device‐measured physical activity, SED, and carotid IMT by addressing a research gap in the young adult population. Previous device‐based evidence among younger individuals was limited to two studies from the European Youth Heart Study—one cross‐sectional study in adolescents [20] and one longitudinal study following participants from childhood to adolescence [19]—as well as one study in the Avon Longitudinal Study of Parents and Children that followed adolescents into young adulthood [21]. However, none of these studies accounted for all 24‐h movement behaviors or their codependency. No cross‐sectional or longitudinal associations between MVPA and carotid IMT were observed across the 6‐year follow‐up among Danish children [19, 20]. In another 13‐year follow‐up study among adolescents aged 11 years from the UK (*n* = 1200) [21], cumulative exposure to LPA and MVPA was associated with lower odds of progressive carotid IMT worsening, although MVPA‐related findings seemed to be present only in women, and more consistent associations were observed for arterial stiffness (carotid‐femoral pulse wave velocity) than for carotid IMT. However, in a cross‐sectional examination of the same cohort at the age of 11 years, no compositional associations were observed between SED, LPA, MVPA, and arterial stiffness, suggesting that such associations may not yet be detectable at younger ages or in cross‐sectional settings, but may emerge and become more apparent over time with growth and development [54]. In addition to methodological differences, for example, in study design (cross‐sectional vs. longitudinal), different findings may be explained by a smaller and possibly healthier study population in the current study (indicated by, e.g., lower mean carotid IMT with a narrower distribution and higher MVPA). The evidence of the association of physical activity and SED with carotid IMT among other age groups remains inconsistent, but longitudinal studies suggest a potential protective role of MVPA [22, 23]. Given the inconsistency of findings across age groups and indications of potential sex differences, more longitudinal investigations are particularly needed to determine how changes in 24‐h movement behaviors associate with arterial IMT progression over time.

In post hoc analyses, we observed an association between higher MVPA and higher systolic blood pressure. Moreover, for LPA, a weak trend towards lower systolic blood pressure was observed with theoretical time reallocation from SED to LPA. However, in this young cohort, these modest blood‐pressure‐related differences were not reflected in IMT measures. The modestly higher systolic blood pressure observed with greater MVPA may partly reflect higher fat‐free body mass and related increases in stroke volume and cardiac output [11, 55].

We were able to examine device‐based sleep as one important component of 24‐h movement behavior composition, and to extend previous evidence on the associations between device‐based sleep and arterial IMT to young adults. We did not find associations between sleep duration and arterial IMT, whereas previous cross‐sectional studies solely focusing on device‐based sleep duration (thus, ignoring other 24‐h movement behaviors) among middle‐aged and older adults have reported that short sleep (< 5 h per night) was associated with higher carotid IMT compared with sleep duration of more than 7 h per night [28], and that longer sleep duration was associated with lower carotid IMT, but only among men [30]. One study also found an indication of a U‐shaped association between sleep duration and carotid IMT, as carotid IMT was reported to be significantly higher among those with less than 5 h of sleep per night and also among those with more than 8 h of sleep per night when compared to those with sleep within the normal range (5–8 h per night) [29]. The U‐shaped association has also been a common finding in the literature concerning cardiometabolic health [50, 51]. We further examined whether sleep duration (< 7 h, 7–9 h, > 9 h) modified the associations between 24‐h movement behaviors and aortic or carotid IMT, but no modifying effects were observed. Given that the number of participants with less than 5 h of sleep was very low, we were not able to use a similar definition of short sleep as used in previous studies on carotid IMT [29]. Future studies with a higher representation of both those with short and long sleep are needed to elaborate on the associations between sleep duration and arterial IMT.

The most important strengths of this study include the use of device‐based measurements of 24‐h movement behaviors, which are less vulnerable to recall bias than self‐report [17], and the in vivo assessment of arterial IMT. Importantly, we were able to examine the associations of 24‐h movement behaviors with rarely assessed aortic IMT—a likely earlier marker of atherosclerotic arterial changes. Moreover, to examine associations between 24‐h movement behaviors and arterial IMT, we applied a state‐of‐the‐art statistical method, CoDA, which addresses the co‐dependency between 24‐h movement behaviors [31, 32, 49].

Our study has some limitations that should be taken into account when interpreting the findings. The cross‐sectional study design prevents us from establishing a causal relationship between 24‐h movement behaviors and arterial IMT. The relatively small study sample increases the risk of Type II errors (failure to identify a significant effect that actually exists) [56], as limited statistical power may have hindered the detection of associations in this young and generally healthy population. Notably, the observed variation in carotid IMT was small, which may have further limited our ability to detect associations for carotid IMT. However, despite the possibility of limited power to detect very small differences, the confidence intervals suggest that, for the examined 30‐min time reallocations, carotid IMT differences exceeding approximately 0.01 mm were generally not supported by the data. Device‐based measurements of 24‐h movement behaviors are subject to measurement error. Wrist‐worn accelerometers have inherent limitations in capturing some types of physical activity, such as cycling and weightlifting, and in separating standing from sitting, which may lead to some inaccuracy in the daily estimates of 24‐h movement behaviors [57]. Moreover, although there were no marked differences in terms of self‐reported physical activity, sitting, or IMT measures between the present study population and those participants who did not provide adequate accelerometer data, possible selection bias should be taken into account when interpreting the findings. Families that originally took part in the trial might have been more interested in health‐related issues than families that did not participate. Furthermore, half of the participants have received systematic counseling on a heart‐healthy diet since infancy, and although the control group children did not receive the dietary counseling, they were probably more aware of their health‐related behaviors than typical Finnish children. Given that belonging to the trial likely has led the STRIP participants to have healthier lifestyles than their peers, our results may underestimate the true associations between 24‐h movement behaviors and arterial IMT.

## 5 Perspective

This study utilized 24‐h accelerometer and ultrasound measurements of both aortic and carotid IMT as well as compositional data analysis to examine combined associations between 24‐h movement behaviors and aortic and carotid IMT among young adults. Although some slight trends towards higher LPA and smaller aortic IMT were found, no significant associations were observed for either aortic or carotid IMT. Nevertheless, exploratory post hoc analyses suggested a weak trend towards lower systolic blood pressure with a theoretical replacement of SED with LPA, consistent with the direction of the trend observed for aortic IMT. Given the growing interest in the health effects of LPA and the limited evidence regarding its role in vascular health, particularly arterial wall structure, future longitudinal and experimental studies are needed to determine how greater LPA is associated with trajectories of systolic blood pressure and arterial IMT. Such studies should consider LPA in the context of the overall 24‐h movement behavior composition, as its potential vascular associations may depend on how time is distributed across sleep, SED, and MVPA.

## Author Contributions

S.R., J.V., T.R., H.N., O.R., and K.P. designed the longitudinal STRIP study, including the follow‐up at age 26 years. J.S.K. performed the arterial I.M.T. measurements. K.P. and S.S. designed the accelerometry data collections and the herein described study. K.S. conducted the analyses with the help of J.P. K.S. drafted the manuscript. All authors contributed to data interpretation, revised the article critically, and approved the final version of the manuscript.

## Funding

This work was supported by the Academy of Finland (grant numbers: 206374, 294834, 251360, 275595, 307996, 322112, and 332030); the Juho Vainio Foundation; the Finnish Foundation for Cardiovascular Research; the Finnish Ministry of Education and Culture; the Finnish Cultural Foundation; the Sigrid Jusélius Foundation; Special Governmental grants for Health Sciences Research, Turku University Hospital; the Yrjö Jahnsson Foundation; the Finnish Medical Foundation; and the Turku University Foundation.

## Ethics Statement

The study has been approved by the Joint Commission on Ethics of Turku University and Turku University Central Hospital (51/1801/2014).

## Consent

Written informed consent was obtained from parents at study entry, and from the participants at the ages of 15, 18, and 26 years.

## Conflicts of Interest

The authors declare no conflicts of interest.

## Supporting information


**Data S1:** Flow chart for the selection of the study population.
**Data S2:** Detailed description of the measurement of arterial IMT at the 26‐year follow‐up point.
**Data S3:** Comparison between the study population included in the analyses (*n* = 327) and those who were not willing to participate in the accelerometer measurement or did not provide adequate accelerometer or arterial IMT data (*n* = 213).
**Data S4:** Associations between the composition of 24‐h movement behaviors (expressed as ilr coordinates) and aortic and carotid intima‐media thickness (IMT).
**Data S5:** Estimated cross‐sectional differences in the odds of elevated aortic intima‐media thickness (IMT) (90th percentile by sex) associated with theoretical 30 min reallocations between 24‐h movement behaviors (e.g., A. from MVPA, LPA, and SED to sleep) relative to the mean 24‐h movement behavior composition in the study population (8.0 h sleep, 12.8 h SED, 1.7 h LPA, and 1.4 h MVPA). Model 1 adjusted for sex and education, Model 2 for sex, education, smoking, and distribution of dietary fats.
**Data S6:** Estimated cross‐sectional differences in the odds of elevated carotid intima‐media thickness (IMT) (90th percentile by sex) associated with theoretical 30 min reallocations between 24‐h movement behaviors (e.g., A. from MVPA, LPA, and SED to sleep) relative to the mean 24‐h movement behavior composition in the study population (8.0 h sleep, 12.8 h SED, 1.7 h LPA, and 1.4 h MVPA). Model 1 adjusted for sex and education, Model 2 for sex, education, smoking, and distribution of dietary fats.
**Data S7:** Associations between the composition of 24‐h movement behaviors (expressed as ilr coordinates) and aortic and carotid intima‐media thickness (IMT) adjusted for accelerometer wear time.
**Data S8:** Associations between the composition of 24‐h movement behaviors (expressed as ilr coordinates) and systolic and diastolic blood pressure (BP).
**Data S9:** Estimated cross‐sectional differences in systolic blood pressure (BP) associated with theoretical one‐to‐one reallocations between 24‐h movement behaviors (e.g., from SED to LPA) relative to the mean 24‐h movement behavior composition in the study population (8.0 h sleep, 12.8 h SED, 1.7 h LPA, and 1.4 h MVPA). Estimates were obtained from a sex‐ and education‐adjusted compositional linear regression model. Shaded areas indicate 95% confidence intervals around the estimated differences.
**Data S10:** Estimated cross‐sectional differences in diastolic blood pressure (BP) associated with theoretical one‐to‐one reallocations between 24‐h movement behaviors (e.g., from SED to LPA) relative to the mean 24‐h movement behavior composition in the study population (8.0 h sleep, 12.8 h SED, 1.7 h LPA, and 1.4 h MVPA). Estimates were obtained from a sex‐ and education‐adjusted compositional linear regression model. Shaded areas indicate 95% confidence intervals around the estimated differences.

## Data Availability

Selected variables and their descriptions without personal identification codes are distributed to investigators and collaborators working on specific projects. The rights to the data belong to the STRIP research group. Data sharing outside the STRIP group requires a data sharing agreement. Investigators can submit an expression of interest to the STRIP Steering Committee.
