# Supplementary material for: Device‐Measured 24‐h Movement Behaviors and Arterial Intima‐Media Thickness in Young Adults
Source: Scand J Med Sci Sports. 2026 Oct 24;36(11):e70387. doi: 10.1111/sms.70387 (PMC13695045; doi:10.1111/sms.70387)
Supplement: Supplementary file 1 [file SMS-36-e70387-s001.docx]

**Supplemental material**

# **Supplement 1.** Flow chart for the selection of the study population.

**Special Turku Coronary Risk Factor Intervention Project (STRIP) follow-up study**

**Study visit at the age of 26 years n=546**

Not willing to participate in the 26-yr accelerometer measurement n=58

Expressed willingness to participate in the 26-yr accelerometer measurement n=488

Did not give contact information for accelerometer delivery n=2

Did not wear the accelerometer at the 26-yr measurement n=84

Wore the accelerometer at 26-yr measurement n=402

Type 1 diabetes n=4*

Had <4 valid measurement days n=58

Device malfunction n=5

Missing data on aortic and/or carotid IMT n=8

**Final analytical sample**

**n=327**

*In total, six participants had type 1 diabetes. Two were excluded at an earlier stage because they did not wear the accelerometer at the 26-yr measurement.

# **Supplement 2.** Detailed description of the measurement of arterial IMT at the 26-year follow-up point.

IMT of the abdominal aorta and common carotid arteries was assessed using a GE Vivid E9 ultrasound system (GE Vingmed Ultrasound A/S, Horten, Norway) according to standardized protocols [1–3]. Ultrasound scans were performed by an experienced sonographer blinded to participant details.

For the assessment of common carotid IMT, both the right and left common carotid arteries were scanned using an 11L linear transducer. After identifying the carotid bifurcation, the image was focused on the common carotid artery 10 mm proximal to the carotid bifurcation, ensuring that both the near and far wall intima were visible. A 5-second moving scan including the common carotid artery and the beginning of the bifurcation was captured and stored for subsequent offline analysis. Common carotid IMT measurements were obtained with a semi-automated TOMTEC AutoIMT software (Version TTA2.41.00., TOMTEC Imaging Systems GmbH, Unterschleissheim, Germany) by a single reader blinded to participant details. The best-quality end-diastolic frame (incident with the R wave on a continuously recorded electrocardiogram) was selected from the 5-second clip. On the selected frame, a region of interest approximately 10 mm wide was drawn 5 to 15 mm proximal to the carotid bifurcation. The software automatically detected the lumen-intima and media-adventitia boundaries of the far wall, traced them, and calculated the mean IMT for the defined region of interest. All automated IMT measurements were reviewed and approved by the reader. The mean of the right and left common carotid IMT values was used for statistical analysis.

For the assessment of aortic IMT, an 11L linear transducer was used to scan the most distal 15 mm of the abdominal aorta proximal to the bifurcation. A scanning frequency of 12 MHz was preferred; however, when necessary (to reach sufficient tissue penetration), lower scanning frequencies, as low as 8 MHz, were used. Gain settings were used to optimize the image quality, and when both the near and far wall intima were visible, the zoom function was applied to magnify a 20 mm segment of the distal abdominal aorta. A moving clip covering at least two QRS complexes on a continuously recorded electrocardiogram was captured and stored for subsequent offline analysis. Aortic IMT was measured by a single reader blinded to participant details. The best-quality end-diastolic frame was selected from the moving clip image. On the selected frame, a region of interest was drawn using TOMTEC software on the section where the IMT was most clearly visualized. The software automatically detected the lumen–intima and media–adventitia boundaries of the aortic far wall, traced these boundaries, and calculated the mean IMT for the defined region of interest. All IMT measurements were reviewed and approved by the reader.

To assess between-observer and between-visit reproducibility of IMT measurements, 50 participants were re-examined. The between-observer coefficient of variation (CV) for IMT measurements was 5.3% for carotid IMT and 5.7% for aortic IMT. The between-visit CV was 3.8% for carotid IMT and 5.0% for aortic IMT.

**References**

1.	Pahkala K, Hietalampi H, Laitinen TT, et al. Ideal cardiovascular health in adolescence: effect of lifestyle intervention and association with vascular intima-media thickness and elasticity (the Special Turku Coronary Risk Factor Intervention Project for Children [STRIP] study). *Circulation*. 2013;127(21):2088–96. doi:10.1161/CIRCULATIONAHA.112.000761

2.	Järvisalo MJ, Jartti L, Näntö-Salonen K, et al. Increased aortic intima-media thickness: a marker of preclinical atherosclerosis in high-risk children. *Circulation*. 2001;104(24):2943–7. doi:10.1161/hc4901.100522

3.	Kallio K, Jokinen E, Saarinen M, et al. Arterial intima-media thickness, endothelial function, and apolipoproteins in adolescents frequently exposed to tobacco smoke. *Circ Cardiovasc Qual Outcomes.* 2010;3(2):196–203. doi:10.1161/CIRCOUTCOMES.109.857771

**Supplement 3.** Comparison between the study population included in the analyses (n=327) and those who were not willing to participate in the accelerometer measurement or did not provide adequate accelerometer or arterial IMT data (n=213).

|  | Analytical sample  N=327 | Participants without accelerometer or IMT data N=213* | Comparison among participants with available data  P value |
| --- | --- | --- | --- |
| Women, N (%) | 195 (60) | 108 (51) | 0.04 |
| Level of education, N (%) |  |  |  |
| Lower | 105 (32) | 66 (31) | 0.82 |
| Higher | 217 (66) | 119 (56) |  |
| Missing | 5 (2) | 28 (13) |  |
| Current smoking, N (%)** | 29 (9) | 23 (11) | 0.22 |
| Distribution of dietary fats, mean (SD)*** | 1.6 (0.6) | 1.5 (0.5) | 0.81 |
| Self-reported LTPA, mean (SD), MET h/week | 25.3 (18.8) | 23.0 (19.0) | 0.19 |
| Self-reported sitting, mean (SD), h/day | 7.1 (2.7) | 7.5 (3.2) | 0.15 |
| Self-reported sleep time, mean (SD), h/night | 7.5 (0.8) | 7.3 (0.9) | 0.005 |
| Aortic IMT, mean (SD), mm | 0.60 (0.15) | 0.61 (0.14) | 0.72 |
| Carotid IMT, mean (SD), mm | 0.46 (0.05) | 0.46 (0.05) | 0.32 |
| BMI, mean (SD), kg/m^2^ | 24.4 (4.1) | 24.5 (4.7) | 0.71 |
| Waist circumference, mean (SD), cm | 80.3 (11.4) | 82.0 (11.2) | 0.09 |
| Systolic blood pressure, mean (SD), mmHg | 120.0 (10.4) | 121.8 (11.2) | 0.05 |
| Diastolic blood pressure, mean (SD), mmHg | 71.6 (6.8) | 72.3 (8.2) | 0.27 |
| Total cholesterol, mean (SD), mmol/L | 4.6 (0.9) | 4.6 (0.9) | 0.72 |
| LDL cholesterol, mean (SD), mmol/L | 2.8 (0.8) | 2.8 (0.7) | 0.45 |
| HDL cholesterol, mean (SD), mmol/L | 1.3 (0.3) | 1.3 (0.3) | 0.91 |
| Triglycerides, mean (SD), mmol/L | 1.0 (0.5) | 1.1 (0.5) | 0.20 |
| HOMA-IR, mean (SD) | 1.7 (1.1) | 1.8 (1.4) | 0.30 |
| hs-CRP, mean (SD), mg/L | 2.0 (3.4) | 2.0 (3.9) | 0.95 |

LTPA = leisure time physical activity, IMT=intima-media thickness, BMI=body mass index, LDL=low-density lipoprotein, HDL=high-density lipoprotein, HOMA-IR=homeostasis model assessment of insulin resistance, hs-CRP=high-sensitivity C-reactive protein

*Those with type 1 diabetes (n=6) were excluded

**Missing values, n=5 (2.0%) among the analytical sample and n=28 (13%) among participants without accelerometer or IMT data. Percentages are based on the total study samples (n=327/213).

*** A ratio of monounsaturated fatty acids (MUFA) and polyunsaturated fatty acids (PUFA) to saturated fatty acids (SFA) ((MUFA+PUFA)/SFA)

**Supplement 4.** Associations between the composition of 24-hour movement behaviors (expressed as ilr coordinates) and aortic and carotid intima-media thickness (IMT).

|  |  | Aortic IMT | | | Carotid IMT | | | | |
| --- | --- | --- | --- | --- | --- | --- | --- | --- | --- |
|  | n | β_ilr_ | 95% CI | p value | β_ilr_ | 95% CI | | p value |  |
| Sleep vs. SED, LPA & MVPA |  |  |  |  |  |  | |  |  |
| Model 1 | 327 | -0.013 | -0.131 to 0.106 | 0.833 | 0.004 | -0.036 to 0.044 | | 0.850 |  |
| Model 2 | 322 | -0.004 | -0.125 to 0.117 | 0.947 | 0.004 | -0.036 to 0.045 | | 0.830 |  |
| Model 3 | 303 | -0.008 | -0.133 to 0.118 | 0.905 | -0.002 | -0.043 to 0.039 | | 0.929 |  |
| Model 4 | 302 | -0.008 | -0.135 to 0.118 | 0.897 | 0.001 | -0.040 to 0.042 | | 0.968 |  |
| SED vs. sleep, LPA & MVPA |  |  |  |  |  |  | |  |  |
| Model 1 | 327 | 0.026 | -0.076 to 0.128 | 0.619 | -0.005 | -0.039 to 0.030 | | 0.790 |  |
| Model 2 | 322 | 0.021 | -0.083 to 0.124 | 0.696 | -0.004 | -0.038 to 0.031 | | 0.840 |  |
| Model 3 | 303 | 0.025 | -0.082 to 0.131 | 0.652 | -0.001 | -0.035 to 0.035 | | 0.998 |  |
| Model 4 | 302 | 0.021 | -0.086 to 0.129 | 0.699 | -0.002 | | -0.037 to 0.033 | 0.903 | |
| LPA vs. sleep, SED & MVPA |  |  |  |  |  |  | |  |  |
| Model 1 | 327 | -0.018 | -0.070 to 0.033 | 0.488 | 0.001 | -0.017 to 0.017 | | 0.985 |  |
| Model 2 | 322 | -0.024 | -0.078 to 0.029 | 0.373 | -0.003 | -0.021 to 0.015 | | 0.723 |  |
| Model 3 | 303 | -0.031 | -0.088 to 0.027 | 0.298 | -0.003 | -0.022 to 0.016 | | 0.766 |  |
| Model 4 | 302 | -0.026 | -0.085 to 0.032 | 0.378 | -0.001 | -0.020 to 0.018 | | 0.922 |  |
| MVPA vs. sleep, SED & LPA |  |  |  |  |  |  | |  |  |
| Model 1 | 327 | 0.005 | -0.047 to 0.058 | 0.849 | 0.001 | -0.017 to 0.018 | | 0.942 |  |
| Model 2 | 322 | 0.008 | -0.046 to 0.062 | 0.770 | 0.002 | -0.016 to 0.020 | | 0.795 |  |
| Model 3 | 303 | 0.014 | -0.042 to 0.070 | 0.631 | 0.005 | -0.014 to 0.023 | | 0.609 |  |
| Model 4 | 302 | 0.014 | -0.043 to 0.070 | 0.637 | 0.002 | -0.016 to 0.021 | | 0.807 |  |

Model 1 adjusted for sex, Model 2 for sex and education, Model 3 for sex, education, smoking, and distribution of dietary fats, Model 4 for sex, education, smoking, distribution of dietary fats, body mass index (BMI), homeostasis model assessment of insulin resistance (HOMA-IR), and systolic blood pressure. β_ilr_ coefficients indicate the change in outcome (mm) for each 1 unit ilr increase, thus pointing to the presence of association, but effect sizes cannot be drawn directly from coefficients.

# **Supplement 5.** Estimated cross-sectional differences in the odds of elevated aortic intima-media thickness (IMT) (90^th^ percentile by sex) associated with theoretical 30 min reallocations between 24-hour movement behaviors (e.g., A. from MVPA, LPA, and SED to sleep) relative to the mean 24-hour movement behavior composition in the study population (8.0 h sleep, 12.8 h SED, 1.7 h LPA, and 1.4 h MVPA). Model 1 adjusted for sex and education, Model 2 for sex, education, smoking, and distribution of dietary fats.

# **Supplement 6.** Estimated cross-sectional differences in the odds of elevated carotid intima-media thickness (IMT) (90th percentile by sex) associated with theoretical 30 min reallocations between 24-hour movement behaviors (e.g., A. from MVPA, LPA, and SED to sleep) relative to the mean 24-hour movement behavior composition in the study population (8.0 h sleep, 12.8 h SED, 1.7 h LPA, and 1.4 h MVPA). Model 1 adjusted for sex and education, Model 2 for sex, education, smoking, and distribution of dietary fats.

**Supplement 7.** Associations between the composition of 24-hour movement behaviors (expressed as ilr coordinates) and aortic and carotid intima-media thickness (IMT) adjusted for accelerometer wear time.

|  | Aortic IMT | | | Carotid IMT | | |
| --- | --- | --- | --- | --- | --- | --- |
|  | β_ilr_ | 95% CI | p value | β_ilr_ | 95% CI | p value |
| Sleep vs. SED, LPA & MVPA | 0.009 | -0.111 to 0.130 | 0.879 | 0.007 | -0.034 to 0.047 | 0.747 |
| SED vs. sleep, LPA & MVPA | 0.010 | -0.094 to 0.113 | 0.855 | -0.005 | -0.040 to 0.029 | 0.761 |
| LPA vs. sleep, SED & MVPA | -0.029 | -0.082 to 0.025 | 0.295 | -0.004 | -0.022 to 0.014 | 0.667 |
| MVPA vs. sleep, SED & LPA | 0.010 | -0.044 to 0.063 | 0.724 | 0.003 | -0.015 to 0.021 | 0.772 |

Adjusted for sex, education and accelerometer wear time. β_ilr_ coefficients indicate the change in outcome (mm) for each 1 unit ilr increase, thus pointing to the presence of association, but effect sizes cannot be drawn directly from coefficients.

**Supplement 8.** Associations between the composition of 24-hour movement behaviors (expressed as ilr coordinates) and systolic and diastolic blood pressure (BP).

|  |  | Systolic BP | | | Diastolic BP | | |
| --- | --- | --- | --- | --- | --- | --- | --- |
|  | n | β_ilr_ | 95% CI | p value | β_ilr_ | 95% CI | p value |
| Sleep vs. SED, LPA & MVPA |  |  |  |  |  |  |  |
| Model 1 | 327 | -3.10 | -9.84 to 3.64 | 0.366 | -3.68 | -8.83 to 1.47 | 0.161 |
| Model 2 | 322 | -3.55 | -10.35 to 3.26 | 0.306 | -3.76 | -8.97 to 1.45 | 0.156 |
| Model 3 | 303 | -3.21 | -10.30 to 3.88 | 0.373 | -3.75 | -9.17 to 1.66 | 0.174 |
| SED vs. sleep, LPA & MVPA |  |  |  |  |  |  |  |
| Model 1 | 327 | 1.94 | -3.86 to 7.74 | 0.511 | 3.42 | -1.01 to 7.85 | 0.130 |
| Model 2 | 322 | 2.26 | -3.56 to 8.09 | 0.445 | 3.46 | -1.00 to 7.91 | 0.128 |
| Model 3 | 303 | 1.99 | -4.04 to 8.01 | 0.517 | 3.47 | -1.14 to 8.07 | 0.140 |
| LPA vs. sleep, SED & MVPA |  |  |  |  |  |  |  |
| Model 1 | 327 | -1.47 | -4.40 to 1.45 | 0.323 | -0.42 | -2.65 to 1.81 | 0.712 |
| Model 2 | 322 | -1.97 | -5.00 to 1.07 | 0.203 | -0.73 | -3.05 to 1.60 | 0.539 |
| Model 3 | 303 | -2.43 | -5.68 to 0.82 | 0.143 | -0.79 | -3.27 to 1.69 | 0.532 |
| MVPA vs. sleep, SED & LPA |  |  |  |  |  |  |  |
| Model 1 | 327 | 2.63 | -0.35 to 5.62 | 0.084 | 0.68 | -1.60 to 2.96 | 0.558 |
| Model 2 | 322 | 3.25 | 0.21 to 6.29 | 0.036 | 1.03 | -1.30 to 3.36 | 0.385 |
| Model 3 | 303 | 3.65 | 0.49 to 6.82 | 0.024 | 1.08 | -1.34 to 3.49 | 0.382 |

Model 1 adjusted for sex, Model 2 for sex and education, Model 3 for sex, education, smoking, and distribution of dietary fats. β_ilr_ coefficients indicate the change in outcome (mmHg) for each 1 unit ilr increase, thus pointing to the presence of association, but effect sizes cannot be drawn directly from coefficients.

# **Supplement 9.** Estimated cross-sectional differences in systolic blood pressure (BP) associated with theoretical one-to-one reallocations between 24-hour movement behaviors (e.g., from SED to LPA) relative to the mean 24-hour movement behavior composition in the study population (8.0 h sleep, 12.8 h SED, 1.7 h LPA, and 1.4 h MVPA). Estimates were obtained from a sex- and education-adjusted compositional linear regression model. Shaded areas indicate 95% confidence intervals around the estimated differences.

# **Supplement 10.** Estimated cross-sectional differences in diastolic blood pressure (BP) associated with theoretical one-to-one reallocations between 24-hour movement behaviors (e.g., from SED to LPA) relative to the mean 24-hour movement behavior composition in the study population (8.0 h sleep, 12.8 h SED, 1.7 h LPA, and 1.4 h MVPA). Estimates were obtained from a sex- and education-adjusted compositional linear regression model. Shaded areas indicate 95% confidence intervals around the estimated differences.
